# Integrative multi-omics identifies MEIS3 as a diagnostic biomarker and immune modulator in hypertrophic cardiomyopathy

**DOI:** 10.3389/fimmu.2025.1675467

**Published:** 2025-10-22

**Authors:** Jinchen He, Zehua Zhou, Dejun Kong, Heng Zhu, Chunmei Liu, Yuyuan Wang, Tianqi Wu, Jinfeng Chen, Yan Liao, Qi Wu

**Affiliations:** ^1^ Department of Cardiology, The Second Affiliated Hospital of Chengdu Medical College, Nuclear Industry 416 Hospital, Chengdu, China; ^2^ Irradiation Preservation and Effect Key Laboratory of Sichuan Province, Chengdu, China; ^3^ Department of Plastic Surgery, The Second Affiliated Hospital of Chengdu Medical College, Nuclear Industry 416 Hospital, Chengdu, China; ^4^ Department of Oncology, The Second Affiliated Hospital of Chengdu Medical College, Nuclear Industry 416 Hospital, Chengdu, China

**Keywords:** hypertrophic cardiomyopathy, MEIS3, biomarker, immune infiltration, transcriptomics, single-cell RNA-seq, ceRNA network, immunoregulation

## Abstract

**Background:**

Hypertrophic cardiomyopathy (HCM) is a prevalent genetic cardiac disorder characterized by myocardial hypertrophy and diastolic dysfunction. While traditionally attributed to sarcomeric mutations, recent studies have highlighted the pivotal contribution of immune dysregulation and stromal–immune interactions in its pathophysiology. However, the molecular drivers bridging structural remodeling and immune activation remain poorly defined.

**Objective:**

This study aimed to characterize the clinical and immunological role of the transcription factor MEIS3 in HCM through integrative transcriptomic and single-cell analyses, with a focus on its diagnostic potential and regulatory interactions within the cardiac microenvironment.

**Methods:**

We performed bulk RNA sequencing on peripheral blood samples from clinically diagnosed HCM patients (n = 4) and matched healthy controls (n = 3), followed by differential expression analysis and weighted gene co-expression network analysis (WGCNA). Machine learning algorithms (LASSO and Random Forest) were used to identify key diagnostic genes. Single-cell RNA sequencing (scRNA-seq) from myocardial tissues was used to localize gene expression. The immunological context was evaluated via xCell-based immune deconvolution, cytokine–immune cell correlation analysis, and ceRNA network construction centered on MEIS3.

**Results:**

MEIS3 was significantly upregulated in HCM samples and identified as a core hub gene in the HCM-associated blue WGCNA module. Machine learning consistently ranked MEIS3 among the top discriminatory markers (AUC > 0.90). scRNA-seq revealed MSCs as the predominant MEIS3-expressing population in HCM myocardium. Functional enrichment implicated MEIS3 in pathways related to protein synthesis, mitochondrial metabolism, and immune modulation. Immune deconvolution indicated increased M1 macrophages, NK cells, and dendritic cells in HCM. MEIS3 expression positively correlated with key immunomodulatory cytokines (CXCL12, BMP1) and altered immune landscapes. The ceRNA network identified candidate lncRNA–miRNA–MEIS3 axes potentially driving its overexpression. Cytokine–immune cell analysis revealed MEIS3-linked cytokines bridging stromal and immune compartments, reinforcing its central role in immunoregulatory remodeling.

**Conclusion:**

MEIS3 functions as a stromal-centric immunomodulator in HCM, shaping cytokine expression and immune infiltration in the diseased heart. Its expression shows diagnostic potential and may represent a novel target for immuno-modulatory strategies. These findings open new avenues for immuno-targeted interventions in HCM management.

## 1 Introduction

Hypertrophic cardiomyopathy (HCM) is a familial heart muscle disease defined by unexplained left ventricular hypertrophy, often leading to heart failure, arrhythmias, or sudden cardiac death in young individuals (1–3). Pathogenic mutations in sarcomeric proteins (e.g., MYH7, MYBPC3) are a well-established cause of HCM, yet a substantial proportion of patients lack detectable sarcomere gene mutations and the genotype-phenotype correlations remain unpredictable. Indeed, up to 50–68% of HCM patients have no identified sarcomere mutation, and the regulatory networks driving HCM pathology in these cases are not fully understood (4, 5). This uncertainty has prompted systematic transcriptome analyses to uncover novel molecular mechanisms and biomarkers that could improve HCM diagnosis and management.

Emerging evidence implicates inflammation and immune dysregulation in HCM progression (6). Although HCM has classically been viewed as a non-inflammatory cardiomyopathy, studies have revealed a chronic, low-grade inflammatory state in HCM patients, characterized by elevated circulating cytokines (e.g., TNF-α, IL-6, IL-1β, IL-10) and mild myocardial immune cell infiltration (7, 8). Histological analyses found that nearly half of septal myectomy samples from HCM patients contain focal accumulations of inflammatory cells (9, 10). Functionally, pro-inflammatory mediators such as TNF-α can drive cardiomyocyte hypertrophy and fibrosis; for example, TNF-α overexpression in myocardium induces IL-6, which in turn promotes hypertrophy, extracellular matrix deposition, and diastolic dysfunction (11, 12). These observations suggest that immune pathways contribute to the HCM phenotype, potentially exacerbating mechanical dysfunction and fibrosis. Consistently, high-throughput studies have identified immune-related signatures in HCM. Gene enrichment analyses show that inflammatory signaling pathways (including MAPK and PI3K–Akt cascades) are upregulated in HCM hearts (13), and immune cell deconvolution has demonstrated increased infiltration of macrophages, monocytes, and NK cells in HCM compared to controls (14). Furthermore, bioinformatic investigations have proposed immune-associated genes as HCM biomarkers. For example, Zheng et al. identified a panel of differentially expressed immune genes that distinguished HCM patients with excellent diagnostic accuracy (C-index 0.925) (15). These findings position the immune response as an important facet of HCM pathogenesis and a potential source of new clinical biomarkers.

Here, we focus on MEIS3 (Meis homeobox 3), a transcription factor not previously linked to HCM, which emerged from our multi-omics analysis as a candidate immunoregulatory gene of interest. MEIS3 belongs to the TALE-homeodomain family of transcription factors known for roles in embryonic development and cell differentiation (16). Notably, MEIS3 can directly regulate PDPK1 (PDK1), a master kinase in the PI3K/Akt signaling pathway, thereby promoting cell survival in other tissues (17). This is intriguing in the context of HCM, where PI3K–Akt signaling and downstream hypertrophic pathways are dysregulated (18). Moreover, recent pan-cancer analyses have revealed that MEIS3 and its family members influence the immune microenvironment (19). High MEIS3 expression in tumors is associated with an “immune-silenced” phenotype characterized by low leukocyte infiltration, and interfering with MEIS family gene expression has been suggested to enhance responses to immunotherapy. While cancer and cardiomyopathy are disparate diseases, these findings hint that MEIS3 might broadly act as an immunomodulatory switch in pathological states. We hypothesized that in HCM, MEIS3 could serve a dual role: as a diagnostic marker reflecting disease status and as an immunoregulatory factor shaping cardiac immune cell engagement and cytokine signaling.

To test this hypothesis, we conducted an integrative analysis combining bulk and single-cell RNA sequencing, machine learning, network biology, and immunoinformatic techniques on myocardial samples from HCM patients and healthy controls. Our study design enabled a comprehensive exploration of MEIS3 from molecular, cellular, and clinical angles. We identified differentially expressed genes and co-expression networks from bulk RNA-seq to pinpoint candidate regulators, and singled out MEIS3 as a top upregulated gene in HCM. We then examined cell type-specific expression of MEIS3 using single-cell transcriptomics to localize its source in cardiac tissue. Immune cell deconvolution (xCell) and cytokine analyses were integrated to determine how MEIS3 expression relates to immune cell infiltration and inflammatory mediator profiles. Furthermore, we constructed a competing endogenous RNA (ceRNA) network to explore upstream lncRNA–miRNA interactions that might regulate MEIS3 in HCM. Finally, we employed machine learning models to evaluate the diagnostic power of MEIS3 (alone and in combination with other features) for distinguishing HCM patients from controls. Through this multi-pronged approach, we uncovered evidence that MEIS3 is intimately linked with the immunopathology of HCM. In this manuscript, we present our findings that highlight MEIS3 as a novel biomarker of HCM and a key node in the immune-related network of disease mechanisms. We discuss the translational implications of targeting MEIS3 or its downstream pathways for improving HCM diagnosis and developing immunomodulatory therapies.

## 2 Materials and methods

### 2.1 Study cohort and blood sample collection

Peripheral blood samples were obtained from 4 patients with obstructive hypertrophic cardiomyopathy (HCM) undergoing septal myectomy (NYHA class III) and 3 healthy donors matched for age and sex. Written informed consent was obtained from all participants, and the study protocol was approved by the institutional ethics committee. Clinical characteristics, including echocardiographic parameters such as maximal left ventricular wall thickness (MLVWT), interventricular septum diameter (IVSd), and posterior wall thickness (LVPWd), were recorded (**Table 1**). None of the HCM patients had known autoimmune or infectious diseases.

**Table 1:** Baseline characteristics of the study subjects.

Patient_ID	Group	Age	Sex	Family_history	NYHA_Class	Apex(mm)	LVPWd(mm)	IVSd(mm)	MLVWT
1	Control	34	F	N	I	7	6	8	8
2	Control	40	M	N	I	8	8	8	8
3	Control	42	M	N	I	8	9	9	9
4	HCM	51	M	N	III	11	11	14	14
5	HCM	55	F	N	III	12	14	16	16
6	HCM	59	M	N	III	13	15	16	16
7	HCM	54	M	N	III	13	13	15	15

### 2.2 RNA extraction and bulk transcriptome profiling

Whole blood was collected in EDTA tubes and processed for RNA extraction using TRIzol reagent. RNA quality was confirmed via Agilent Bioanalyzer (RIN > 7). Poly-A mRNA was enriched for cDNA library construction and sequenced on the Illumina NovaSeq platform (150 bp paired-end reads, ~50M reads/sample). Reads were quality filtered (fastp) and aligned to the human genome (GRCh38) using STAR. Gene-level counts were quantified with featureCounts. Differential expression analysis between HCM and control groups was performed using DESeq2, applying FDR < 0.05 and |log_2_FC| > 1. GO and KEGG enrichment analyses were conducted using the clusterProfiler package to interpret functional significance.

### 2.3 Weighted gene co-expression network analysis

We used the top 5,000 most variable genes to construct a scale-free network in the WGCNA package (R). A soft-thresholding power (β = 8) was chosen, and dynamic tree cutting was applied to detect modules. Module eigengenes were correlated MLVWT. The turquoise module, enriched for immune-related genes, showed the strongest correlation with HCM (r = 0.94), and included MEIS3 as a hub gene.

### 2.4 Functional enrichment analysis

To interpret the biological roles of the key HCM-associated genes, we performed functional enrichment analyses on the overlapping gene set from the DEG and WGCNA intersection. This intersection step was designed to highlight genes that were not only differentially expressed but also embedded within disease-related co-expression modules, thereby increasing biological relevance and reducing potential false positives. Enrichment analyses were carried out using clusterProfiler in R and the DAVID online tool for verification, covering Gene Ontology (GO), Kyoto Encyclopedia of Genes and Genomes (KEGG) pathways, and Disease Ontology (DO) annotations.

### 2.5 Machine learning-based feature selection

We applied two supervised machine learning algorithms—LASSO regression and Random Forest classification—to normalized RNA-seq expression matrices. LASSO logistic regression was implemented using the glmnet package, applying L1 regularization to select a minimal set of informative genes. Model tuning was performed via 10-fold cross-validation to determine the optimal penalty parameter. Genes with non-zero coefficients were retained as key predictors. Random Forest analysis was conducted using the randomForest package to rank genes by variable importance. Feature ranking was based on mean decrease in accuracy and Gini impurity. Genes prioritized by both methods were selected for further validation and interpretation.

### 2.6 Gene set enrichment analysis

Gene Set Enrichment Analysis (GSEA) was performed using the *clusterProfiler* package in R. Genes were ranked by log2 fold-change values between HCM and control samples. Enrichment was assessed against Kyoto Encyclopedia of Genes and Genomes (KEGG) gene sets obtained from the Molecular Signatures Database (MSigDB). Statistical significance was determined using 1,000 permutations, and enrichment scores were reported as normalized enrichment scores (NES). Pathways were considered enriched under the thresholds |NES| > 1, nominal p < 0.05, and false discovery rate (FDR) < 0.25.

### 2.7 Gene set variation analysis

Gene Set Variation Analysis (GSVA) was conducted using the *GSVA* package in R. Normalized expression data were transformed into pathway enrichment scores for each sample using KEGG pathway gene sets. Group-level comparisons between HCM and controls were performed using the *limma* package. Statistical significance was defined as adjusted p < 0.05.

### 2.8 Validation by qPCR

To validate the RNA-seq findings, the expression of the four key genes (MEIS3, SYDE2, TRAT1, ANKRD20A1) was quantified by qPCR in peripheral blood samples from 8 HCM patients and 8 matched healthy controls. cDNA was synthesized from RNA of each sample using a reverse transcription kit. Gene-specific primers were designed spanning exon-exon junctions to ensure specificity to cDNA (primer sequences are listed in **Supplementary Table S1**). qPCR was performed on a real-time PCR system using SYBR Green detection. Each reaction was run in triplicate, and the relative expression of target genes was calculated by the 2^–ΔΔCt^ method, normalizing to a housekeeping gene (GAPDH).

### 2.9 Validation of hub gene expression using external GEO datasets

To independently validate the expression patterns of the identified hub genes, transcriptomic data were retrieved from the Gene Expression Omnibus (GEO) database (GSE249925), which includes myectomy samples from 97 obstructive HCM patients and 23 controls. The Raw expression data were downloaded and processed using the R software environment (version 4.3.1) as standard procedure. To further assess the diagnostic performance of these hub genes, receiver operating characteristic (ROC) curve analyses were performed using the pROC package in R. The area under the curve (AUC) values were calculated to quantify the discriminatory ability.

### 2.10 Single-cell RNA-seq analysis

To identify MEIS3 expression at single-cell resolution, we analyzed publicly available myocardial scRNA-seq data from Figshare (https://doi.org/10.6084/m9.figshare.c.5777948.v2), which consists of 10 HCM patients and 2 healthy donors. The dataset was processed using CellRanger and Seurat (v4.0). Cells with <200 genes or >10% mitochondrial content were filtered. After normalization and integration, clustering and UMAP dimensionality reduction were performed. Cell types were annotated using canonical markers. MEIS3 expression was evaluated across clusters, revealing enrichment in fibroblast-like stromal cells in HCM myocardium.

### 2.11 Immune infiltration estimation with xCell

To profile immune and stromal composition, we applied xCell to normalized bulk RNA-seq data. xCell scores for 64 immune/stromal cell types were compared between HCM and control samples using Welch’s t-test. MEIS3 expression was correlated with selected cell types using Spearman correlation. Samples with higher MEIS3 showed increased fibroblast and smooth muscle signatures and displayed distinct cytokine–cell score associations.

### 2.12 Cytokine–cell correlation analysis

We selected 24 cytokine-related genes and calculated their Pearson correlation with xCell-derived immune/stromal scores. Pearson correlation coefficients were calculated between the expression of each cytokine gene and xCell-derived immune/stromal scores. Analyses were performed in R (v4.3.1), and p-values were adjusted for multiple testing using the Benjamini–Hochberg method.

### 2.13 ceRNA network construction

To investigate post-transcriptional regulation, we predicted MEIS3-targeting miRNAs via miRanda, TargetScan, and Diana_microT. Upregulated lncRNAs were then screened for miRNA response elements using the ENCORI database (via the AGO‐Clip lncRNA prediction tool: https://rnasysu.com/encori/agoClipRNA.php?source=lncRNA). A ceRNA network was constructed with lncRNAs, miRNAs, and MEIS3, suggesting that lncRNA–miRNA competition may underlie MEIS3 dysregulation.

### 2.14 Statistical analysis

All statistical analyses were conducted in R v4.1.0. Group comparisons used Welch’s t-test; correlations were evaluated using Pearson or Spearman methods. P-values were adjusted with the Benjamini–Hochberg method, and significance was defined as P < 0.05.

## 3 Results

### 3.1 Identification of differentially expressed genes in HCM

**Figure 1:**
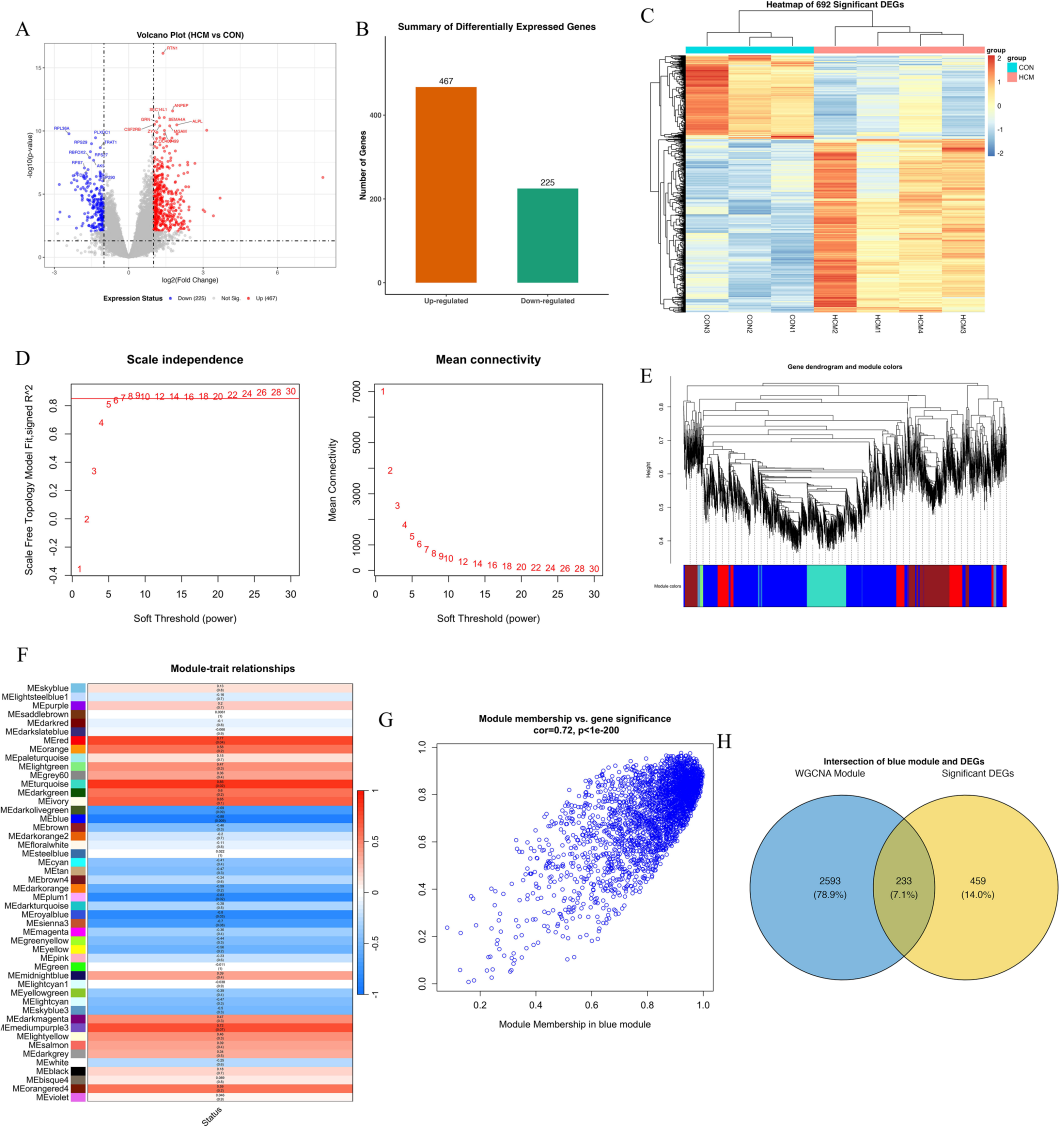
Transcriptomic analysis and WGCNA reveal a key gene module associated with HCM. **(A)** Volcano plot of differentially expressed genes (DEGs) between HCM and control samples. Each point represents a gene; red points indicate significantly upregulated genes (n = 467), blue points indicate downregulated genes (n = 225), and grey points are non-significant. Vertical dashed lines mark the |log_2_ fold change| = 1 threshold; the horizontal dashed line indicates adjusted p = 0.05. **(B)** Bar chart summarizing the number of upregulated (orange) and downregulated (green) DEGs. **(C)** Heatmap of the top 692 DEGs, clustered by gene expression. Rows represent genes, and columns represent individual samples (CON1–3, HCM1–4). **(D)** Determination of soft-thresholding power (β) for WGCNA. Left: Scale-free topology fit index (y-axis) across powers (x-axis), with β = 8 selected where R² > 0.85. Right: Mean connectivity analysis showing a decreasing trend as β increases. **(E)** Gene dendrogram generated from hierarchical clustering based on topological overlap, with dynamic tree cutting identifying multiple co-expression modules (color-coded below dendrogram). **(F)** Heatmap of module–trait correlations. Each cell represents the Pearson correlation coefficient between module eigengene and HCM status, with p-values in parentheses. The MEturquoise module shows the strongest positive correlation with HCM (r = 0.85, p < 0.001). **(G)** Scatter plot showing module membership vs. gene significance within the blue module. Each dot represents a gene; a strong positive correlation (r = 0.72, p < 1e–200) indicates module robustness and relevance to HCM phenotype. **(H)** Venn diagram showing overlap between DEGs and blue module genes. Among 2,826 blue module genes and 692 DEGs, 233 genes are shared (7.1%), representing potential hub candidates.

To investigate transcriptomic alterations in hypertrophic cardiomyopathy (HCM), we performed RNA sequencing of blood samples from HCM patients and matched healthy controls. Principal component analysis (PCA) clearly distinguished the HCM samples from controls, indicating high data quality and distinct phenotypic differences (**Supplementary Figure S1A**). Sample-to-sample correlation analysis further validated strong intra-group similarity and distinct inter-group differences, providing additional support for robust transcriptomic characterization (**Supplementary Figure S1B**). A total of 692 significant DEGs were identified using stringent criteria, comprising 467 up-regulated and 225 down-regulated genes (**Figures 1A, B**). Hierarchical clustering and heatmap visualization further demonstrated distinct gene expression profiles segregating clearly between HCM patients and controls, suggesting robust transcriptomic differences related to HCM pathology (**Figure 1C**).

### 3.2 WGCNA analysis and intersection with DEGs

To identify gene modules associated with the clinical phenotype of HCM, weighted gene co-expression network analysis (WGCNA) was conducted. An optimal soft-threshold power of 8 was selected based on achieving a scale-free topology index above 0.85 and maintaining appropriate mean connectivity (**Figure 1D**). Hierarchical clustering produced multiple gene modules, visualized as a dendrogram with color-coded assignments (**Figure 1E**). The module–trait relationship heatmap highlighted that the turquoise module was most positively associated with HCM (r = 0.94, p < 0.001), whereas the blue module was strongly negatively correlated (r ≈ −0.88, p < 0.001)(**Figure 1F**). Module membership and gene significance analysis further underscored the relevance of the blue module (correlation = 0.72, p < 1e-200; **Figure 1G**). The eigengene adjacency heatmap confirmed robust inter-module relationships, supporting network stability (**Supplementary Figure S1C**). Finally, Venn analysis identified 233 overlapping genes between the blue module and DEGs, suggesting potential regulatory hubs in HCM pathogenesis (**Figure 1H**).

### 3.3 Functional enrichment analyses reveal key biological pathways in HCM

**Figure 2:**
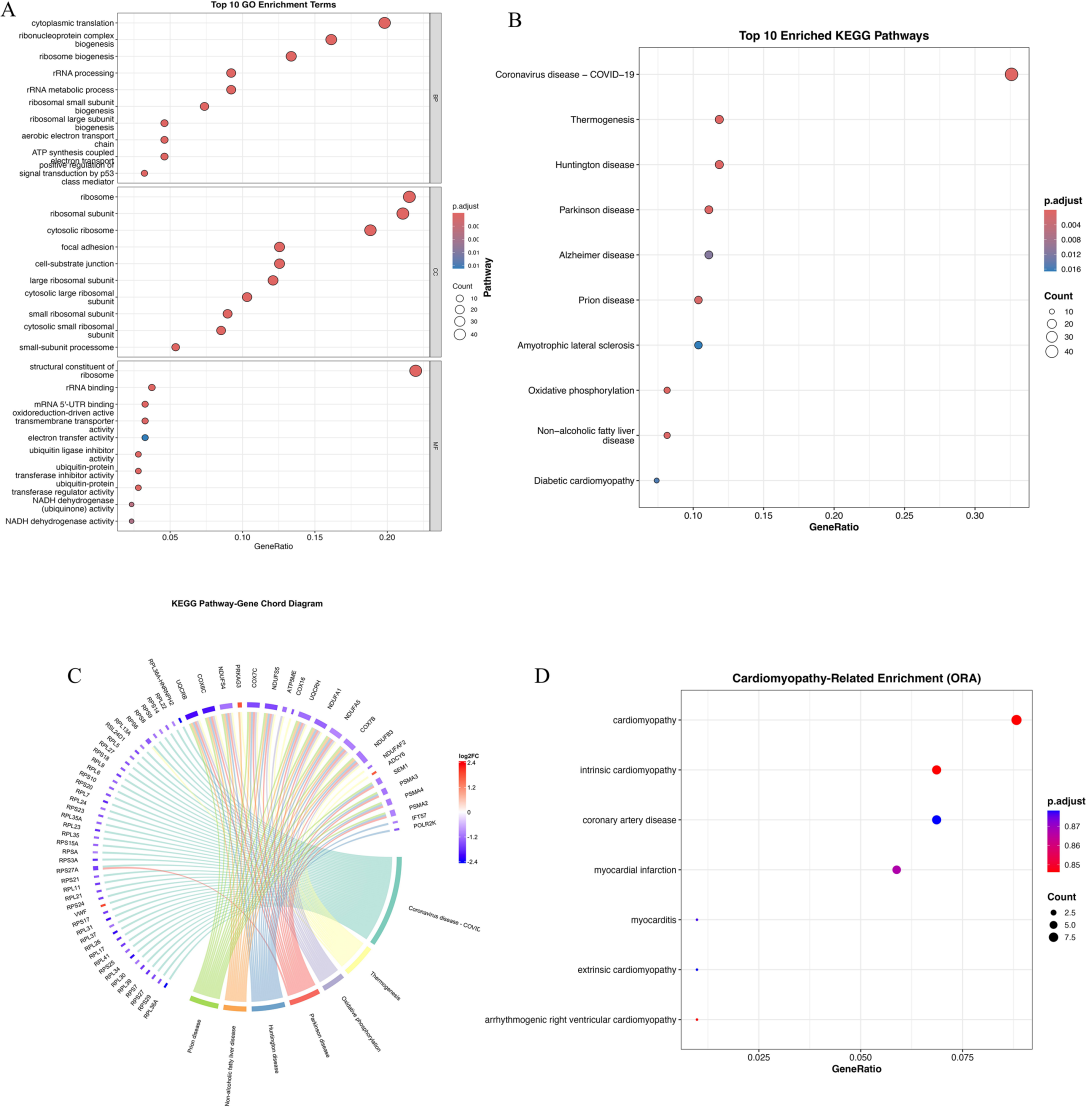
Functional enrichment analysis of overlapping genes from DEGs and WGCNA blue module. **(A)** Gene Ontology (GO) enrichment analysis of overlapping genes categorized into three domains: biological processes (BP), cellular components (CC), and molecular functions (MF). Dot size represents gene count; color gradient reflects adjusted p-value. **(B)** Kyoto Encyclopedia of Genes and Genomes (KEGG) enrichment analysis of top 20 pathways. Notable enriched pathways include thermogenesis, oxidative phosphorylation, Huntington’s disease, Parkinson’s disease, Alzheimer’s disease, and diabetic cardiomyopathy. **(C)** KEGG pathway–gene chord diagram depicting associations between enriched pathways and key genes. Several genes (e.g., NDUFB6, NDUFA9, RPL15) appear in multiple pathways, indicating central regulatory roles in mitochondrial bioenergetics and stress signaling. **(D)** Disease Ontology (DO) enrichment analysis highlighting cardiomyopathy-related terms. Significant enrichment was observed for intrinsic cardiomyopathy, coronary artery disease, myocardial infarction, and myocarditis, indicating disease specificity of the overlapping gene set. Dot color denotes p-value, and size indicates gene count.

To functionally characterize the 233 overlapping genes between DEGs and the WGCNA blue module (**Figure 1H**), we performed GO, KEGG, and DO enrichment analyses. GO terms were enriched in processes related to protein synthesis (*cytoplasmic translation*, *ribosome biogenesis*), mitochondrial structure (*mitochondrial inner membrane*), and redox regulation (*oxidoreductase activity*), indicating altered translational and metabolic states in HCM (**Figure 2A**). KEGG analysis further highlighted pathways such as *thermogenesis*, *oxidative phosphorylation*, and *neurodegenerative disease signaling*, underscoring mitochondrial dysfunction and energy imbalance as potential disease mechanisms (**Figure 2B**). Chord mapping revealed core genes (e.g., *NDUFB6*, *RPL15*, *ATP5PB*) involved in multiple interconnected pathways (**Figure 2C**).DO analysis confirmed enrichment in *cardiomyopathy*, *coronary artery disease*, and *myocardial infarction*, supporting the cardiovascular specificity of the identified genes (**Figure 2D**). Together, these findings underscore the biological significance of the blue module, as its enriched gene set converges on key processes such as mitochondrial energy metabolism, translational regulation, and cardiomyopathy-related pathways. The integrative enrichment analysis not only reinforces the pathological relevance of these genes in HCM but also highlights their potential as mechanistic markers and promising therapeutic targets for disease modulation.

### 3.4 Machine learning-based identification of key genes

**Figure 3:**
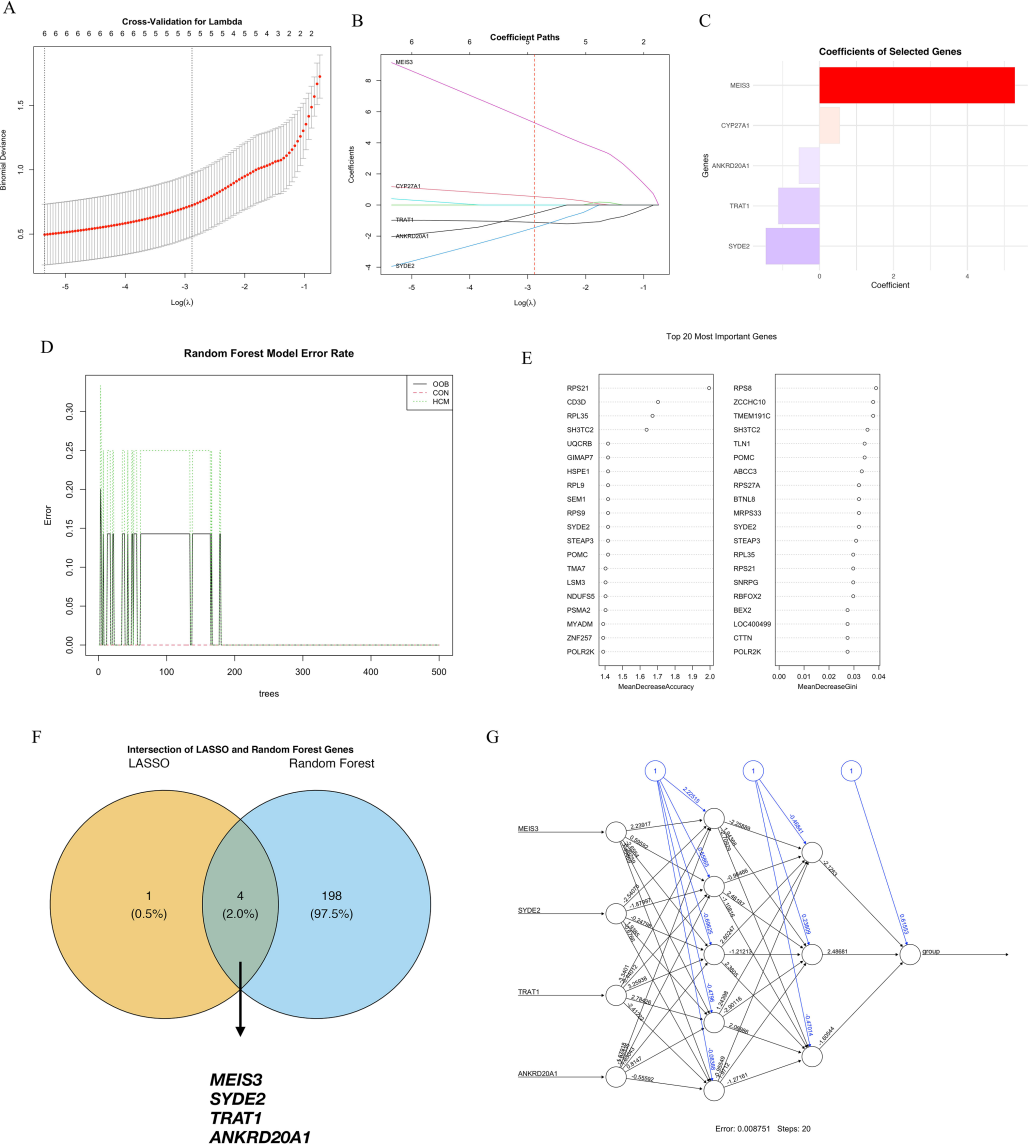
Machine learning-based identification of key diagnostic genes for hypertrophic cardiomyopathy (HCM). **(A)** Ten-fold cross-validation curve for the LASSO logistic regression model used to select the optimal regularization parameter (λ); the dotted line on the left indicates the λ value with minimum mean cross-validated error. **(B)** Coefficient paths of selected genes across varying λ values; five genes retained non-zero coefficients at the optimal λ, including MEIS3, CYP7A1, ANKRD20A1, TRAT1, and SYDE2. **(C)** Bar plot showing the absolute coefficients of LASSO-selected genes, with MEIS3 exhibiting the highest predictive weight. **(D)** Error rates of the Random Forest classifier for HCM, control, and overall samples stabilized after ~150 trees, indicating good model convergence. **(E)** Feature importance scores of the top 20 genes in the Random Forest model ranked by mean decrease in accuracy (left) and Gini index (right). **(F)** Venn diagram showing the intersection between LASSO and Random Forest outputs; four overlapping genes—MEIS3, SYDE2, TRAT1, and ANKRD20A1—were identified as robust diagnostic candidates. **(G)** Neural network topology using these four genes as input nodes, achieving a final error rate of 0.00751 after 20 training steps, supporting their discriminative potential in HCM classification.

To further pinpoint critical genes contributing to HCM, we employed machine learning approaches, namely Least Absolute Shrinkage and Selection Operator (LASSO) and Random Forest algorithms. LASSO regression with 10-fold cross-validation determined the optimal penalty parameter, yielding a parsimonious set of genes with non-zero coefficients, including *MEIS3, CYP7A1, ANKRD20A1, TRAT1*, and *SYDE2* (**Figures 3A–C**). *MEIS3* consistently exhibited the strongest predictive weight. Parallelly, Random Forest analysis demonstrated stable classification error rates after ~150 trees, ranking the top genes by mean decrease accuracy and Gini index (**Figures 3D, E**). Intersection analysis of both methods identified four consistently prioritized genes—*MEIS3, SYDE2, TRAT1*, and *ANKRD20A1*—highlighting their reproducibility and biological relevance (**Figure 3F**). To further validate their discriminative capacity, a feedforward neural network incorporating these four genes was trained, achieving a minimal classification error (0.00751) (**Figure 3G**). Together, these findings underscore *MEIS3, SYDE2, TRAT1*, and *ANKRD20A1* as stable diagnostic candidates, with *MEIS3* emerging as the most prominent.

### 3.5 Pathway enrichment analysis of machine learning-derived candidate genes

We profiled pathway context for the four machine learning–prioritized genes (*MEIS3, SYDE2, TRAT1, ANKRD20A1*) using Gene Set Enrichment Analysis (GSEA) and Gene Set Variation Analysis (GSVA) based on KEGG pathway gene sets.

**Figure 4:**
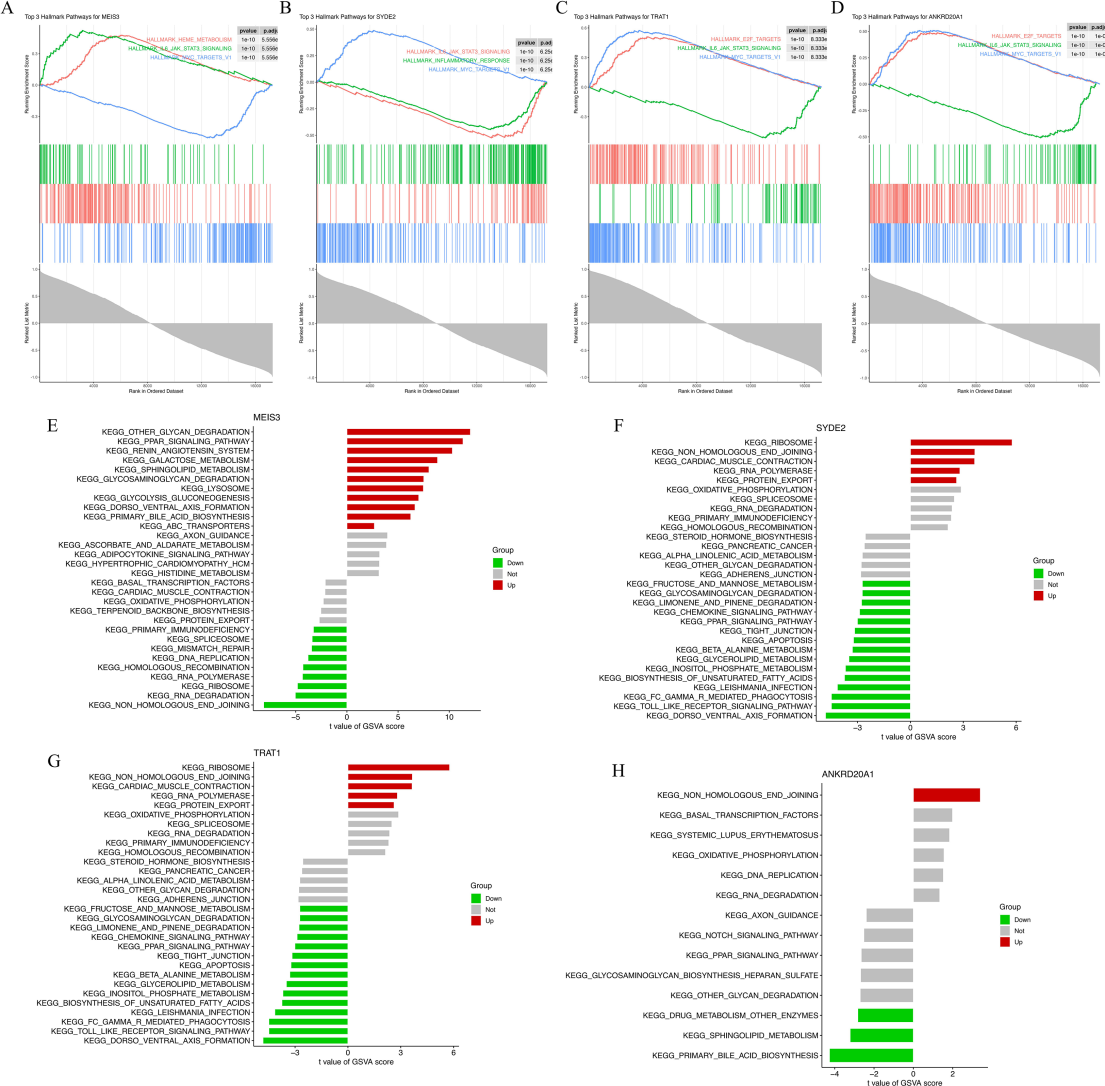
Functional enrichment analysis of four machine learning-derived HCM marker genes. **(A–D)** Gene Set Enrichment Analysis (GSEA) plots for MEIS3 **(A)**, SYDE2 **(B)**, TRAT1 **(C)**, and ANKRD20A1 **(D)** based on KEGG gene sets. Enrichment scores (ES) are shown with leading-edge subsets and normalized enrichment scores (NES) indicated for each gene. **(E–H)** Gene Set Variation Analysis (GSVA) results depicting pathway-level t-values between HCM and control groups for MEIS3 **(E)**, SYDE2 **(F)**, TRAT1 **(G)**, and ANKRD20A1 **(H)**. Red and green bars represent significantly up- or down-regulated pathways, respectively, with gray indicating nonsignificant differences. Pathways span categories including ribosome biogenesis, cardiac muscle contraction, glycan degradation, immune modulation, and oxidative phosphorylation.

GSEA results revealed that MEIS3 was enriched in pathways related to ribosome, glycan degradation, and metabolic signaling (**Figure 4A**). Similarly, SYDE2 showed positive enrichment in calcium signaling, cardiac muscle contraction, and oxidative phosphorylation pathways, which are closely linked to cardiac metabolism and excitation–contraction coupling (**Figure 4B**). TRAT1 was involved in ribosome biogenesis and cardiac-related pathways such as dilated cardiomyopathy (**Figure 4C**), whereas ANKRD20A1 was associated with gene replication and cell cycle-related processes (**Figure 4D**), highlighting its potential role in transcriptional regulation in HCM.

Complementary GSVA analysis provided individual-level pathway activity scores across samples. MEIS3 displayed significantly increased activity in ribosome-related and glycosaminoglycan degradation pathways, whereas immune-related pathways (e.g., JAK-STAT and cytokine signaling) were downregulated in the HCM group (**Figure 4E**). SYDE2 was linked to protein folding, apoptosis, and immune signaling, showing upregulation in metabolic stress pathways (**Figure 4F**). TRAT1 shared several overlapping pathways with SYDE2, while ANKRD20A1 primarily affected DNA replication, transcription factor activity, and systemic lupus erythematosus-related processes (**Figures 4G, H**). Together, these findings support the functional relevance of the four candidate genes in processes spanning protein synthesis, immune regulation, and mitochondrial metabolism—pathways consistent with HCM molecular pathology.

### 3.6 Clinical validation and correlation analysis of key genes

**Figure 5:**
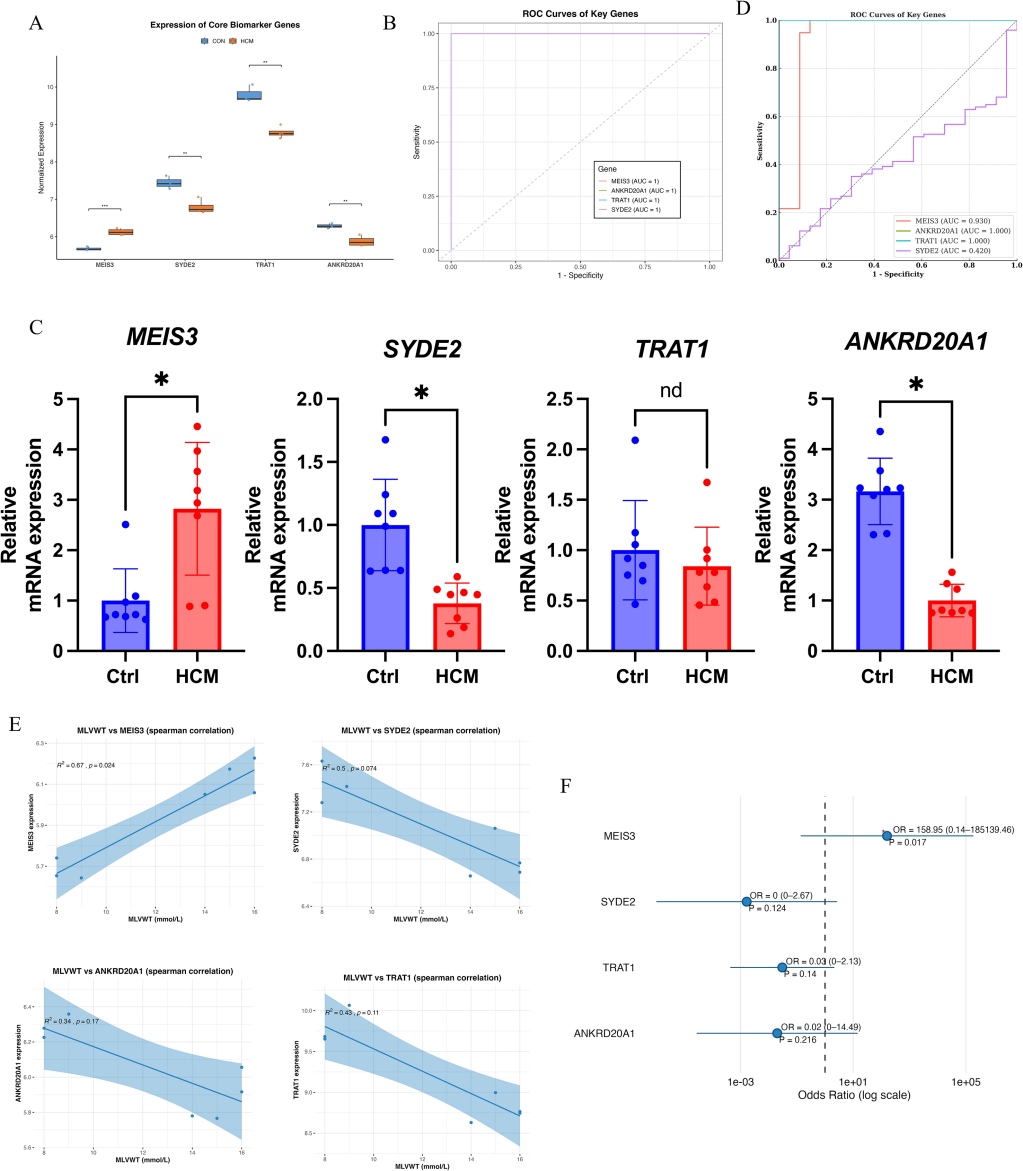
Clinical validation and diagnostic evaluation of key HCM-related genes. **(A)** Boxplot comparing normalized RNA-seq expression levels of four machine learning–selected genes (MEIS3, SYDE2, TRAT1, ANKRD20A1) between control and HCM groups. MEIS3 expression was significantly upregulated in HCM, while SYDE2, TRAT1, and ANKRD20A1 were downregulated. **(B)** Receiver Operating Characteristic (ROC) curve analysis demonstrated excellent classification performance for all four genes, each achieving an area under the curve (AUC) of 1.0, indicating high diagnostic potential. **(C)** qRT-PCR validation in an independent cohort confirmed significantly increased expression of MEIS3 and decreased expression of SYDE2 and ANKRD20A1 in HCM patients (n = 8) compared to controls (n = 8); TRAT1 expression did not differ significantly, suggesting transcript-level variation not reflected at the protein-coding level. **(D)** ROC curve validation of hub genes in the external GEO dataset (GSE249925). **(E)** Spearman correlation analysis between gene expression and maximal left ventricular wall thickness (MLVWT), a key clinical parameter of HCM severity (as shown in **Table 1**), revealed significant positive correlations for MEIS3 (r = 0.67, p = 0.024) and SYDE2 (r = 0.5, p = 0.074), suggesting a possible link with structural myocardial remodeling. ANKRD20A1 and TRAT1 showed non-significant negative trends. **(F)** Forest plot of odds ratio analysis showed that MEIS3 expression was strongly associated with increased HCM risk (OR = 158.95, p = 0.017), highlighting its clinical relevance as a robust biomarker. Other genes showed non-significant associations (p > 0.1), underscoring MEIS3 as the most reliable diagnostic candidate among the four. * indicated P<0.05.

Normalized RNA-seq data confirmed distinct expression patterns for the four candidates: MEIS3 was significantly upregulated in HCM, whereas SYDE2, TRAT1, and ANKRD20A1 were reduced relative to controls (**Figure 5A**). ROC analyses showed strong discrimination for all four genes in the discovery cohort, with AUCs approaching 1.0, further substantiating their potential diagnostic utility (**Figure 5B**). As an orthogonal assay, qPCR in an independent set of peripheral blood samples replicated increased MEIS3 and decreased SYDE2 and ANKRD20A1; TRAT1 did not differ significantly by qPCR (**Figure 5C**). In an external GEO cohort (GSE249925), ROC analysis demonstrated AUC = 1.000 for ANKRD20A1 and TRAT1, AUC ≈ 0.930 for MEIS3, and AUC ≈ 0.420 for SYDE2 (**Figure 5D**). These results confirm the robustness of MEIS3, ANKRD20A1, and TRAT1 as potential diagnostic biomarkers for HCM, while suggesting a weaker role for SYDE2.

To explore their clinical relevance, we investigated the correlation between gene expression levels and maximal left ventricular wall thickness (MLVWT). Among all clinical variables recorded in **Table 1** (including age, sex, NYHA classification, ejection fraction, and interventricular septal thickness), MLVWT was selected due to its strong pathophysiological link to myocardial hypertrophy and remodeling. Spearman correlation analysis showed a significant positive association between MLVWT and MEIS3 (Spearman’s ρ = 0.67, p = 0.024). For SYDE2, the correlation with MLVWT was positive but did not reach statistical significance (Spearman’s ρ = 0.50, p = 0.074). Conversely, ANKRD20A1 and TRAT1 exhibited weaker and non-significant correlations with MLVWT, suggesting differential clinical relevance among these key genes (**Figure 5E**). Finally, odds-ratio analysis indicated that higher MEIS3 expression was associated with increased odds of HCM(**Figure 5F**). Collectively, these findings nominate MEIS3, together with ANKRD20A1 and TRAT1, as promising diagnostic candidates; however, clinical utility will require validation in larger, prospective cohorts and evaluation alongside standard risk markers.

### 3.7 Single-cell RNA-seq analysis validates MEIS3 expression in MSCs and cell-type specific signatures

**Figure 6:**
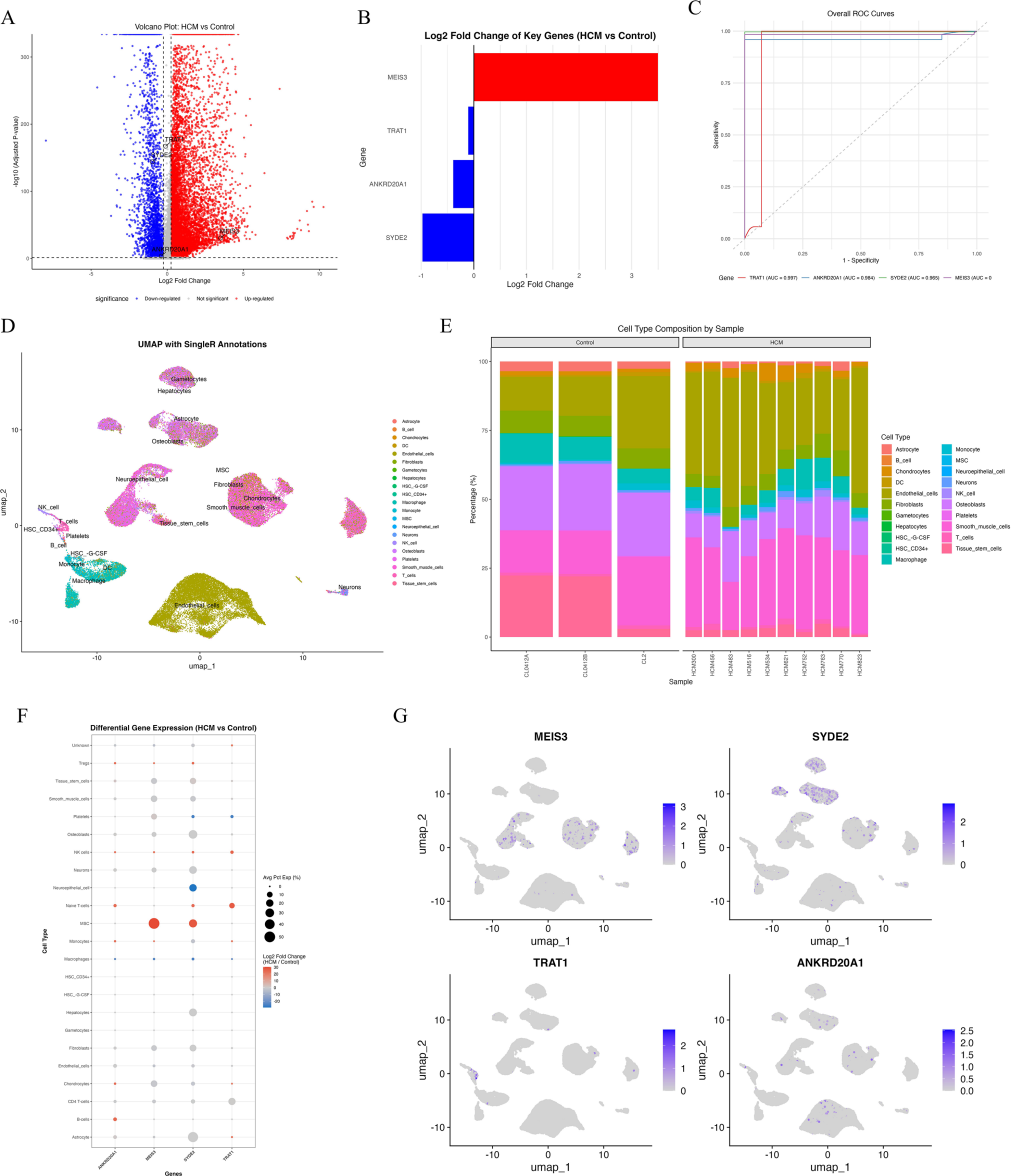
Single-cell transcriptomic analysis reveals cell-type–specific expression patterns of key diagnostic genes in HCM. **(A)** Volcano plot showing differentially expressed genes in scRNA-seq data comparing HCM versus control samples. MEIS3 is significantly upregulated (red), while SYDE2, TRAT1, and ANKRD20A1 are downregulated (blue). **(B)** Bar graph showing log2 fold changes of the four key genes between HCM and control samples. **(C)** ROC curves based on scRNA-seq expression data demonstrating excellent discriminatory performance of MEIS3 among the four genes (AUC ≈ 1.0). **(D)** UMAP plot with SingleR-based cell-type annotation, depicting distinct clustering of major immune and stromal cell populations including monocytes, T/NK cells, fibroblasts, endothelial cells, and mesenchymal stem cells (MSCs). **(E)** Bar plot showing cell-type composition per sample. A noticeable increase in MSC proportion is observed in HCM samples compared to controls. **(F)** Dot plot of cell-type–specific differential expression. MEIS3 is selectively upregulated in MSCs, while SYDE2, TRAT1, and ANKRD20A1 are generally downregulated in multiple cell types including fibroblasts, macrophages, and monocytes. Dot size represents percentage of expressing cells; color denotes log2 fold change. **(G)** UMAP feature plots for MEIS3, SYDE2, TRAT1, and ANKRD20A1. MEIS3 expression is predominantly localized to the MSC cluster, whereas the other three genes show low and scattered expression across cell populations.

Single-cell RNA sequencing (scRNA-seq) confirmed the expression patterns identified in bulk analyses: MEIS3 was significantly upregulated in HCM, while SYDE2, TRAT1, and ANKRD20A1 were downregulated (**Figures 6A, B**). ROC analysis based on scRNA-seq data demonstrated strong diagnostic performance of MEIS3 across cell types (**Figure 6C**). UMAP clustering with SingleR annotation identified major cardiac and immune populations, including monocytes, endothelial cells, fibroblasts, T cells, and mesenchymal stem cells (MSCs) (**Figure 6D**). Notably, MSCs were expanded in HCM compared to controls (**Figure 6E**), indicating potential disease-related remodeling. Cell-type–specific differential expression showed that MEIS3 was selectively enriched in MSCs, whereas the other three candidate genes were broadly suppressed across multiple stromal and immune subsets (**Figure 6F**). UMAP feature mapping further confirmed MEIS3 localization within the MSC compartment (**Figure 6G**). These results highlight MSCs as the principal source of MEIS3 in HCM and suggest its potential role in MSC-driven remodeling processes.

### 3.8 MEIS3-associated cytokine landscape in MSCs

**Figure 7:**
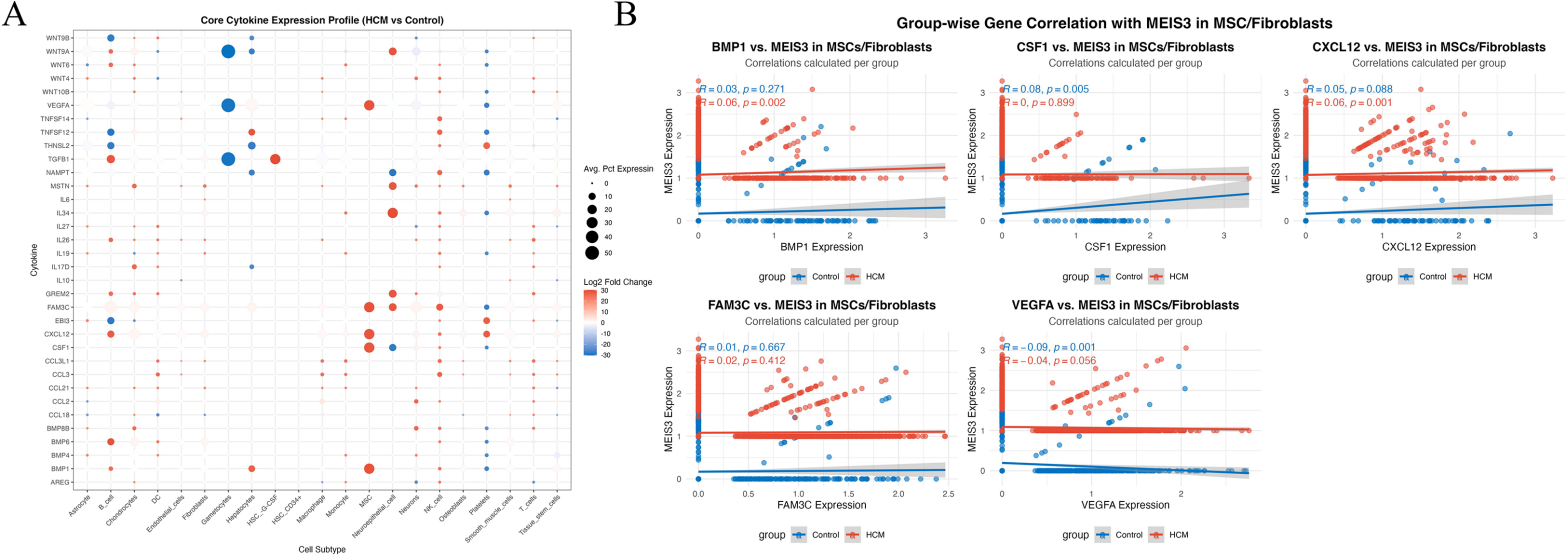
Cytokine expression landscape and MEIS3-associated signaling interactions in MSCs/fibroblasts. **(A)** Dot plot illustrating the average expression and log2 fold change of 40 key cytokines across 21 annotated cell types in HCM versus control conditions. Each dot represents the mean expression percentage (dot size) and direction of differential expression (color scale: red = upregulated; blue = downregulated). Notably, MEIS3-enriched mesenchymal stem cells (MSCs) and fibroblasts displayed pronounced dysregulation of cytokines such as BMP1, CXCL12, CSF1, and VEGFA. **(B)** Group-wise correlation analyses between MEIS3 expression and representative cytokines in MSCs/fibroblasts. Spearman correlation coefficients (r) and p-values are shown separately for control (blue) and HCM (red) groups. MEIS3 expression in HCM MSCs positively correlated with BMP1 (r = 0.06, *p* = 0.002) and CXCL12 (r = 0.06, *p* = 0.001), while an inverse association was observed with VEGFA (r = –0.04, *p* = 0.056). No significant correlations were found for FAM3C or CSF1 within the HCM group. These results suggest that MEIS3 may participate in fibroblast/MSC-mediated paracrine regulation in HCM through selective cytokine interaction.

Building on the selective upregulation of MEIS3 in MSC populations, we profiled cytokine transcripts across MSC and fibroblast subsets to assess potential microenvironmental effects. Dot-plot visualization indicated dysregulation of several stromal/angiogenic and immunoregulatory factors in HCM-derived MSCs relative to controls, including CXCL12, VEGFA, CSF1, and BMP1, which are implicated in angiogenesis, immune-cell recruitment, and extracellular matrix remodeling (**Figure 7A**). These patterns are consistent with a shift toward stromal activation and altered immune signaling in HCM.

To further dissect MEIS3-associated regulatory patterns, we performed group-wise gene correlation analyses between MEIS3 and selected cytokines in MSC/fibroblast populations. MEIS3 expression exhibited statistically detectable but very weak positive correlations with CXCL12 (r = 0.06, p = 0.001) and BMP1 (r = 0.06, p = 0.002), whereas these associations were absent or weaker in controls (**Figure 7B**). Interestingly, VEGFA exhibited a weak negative correlation trend with MEIS3 in HCM, potentially reflecting a compensatory angiogenic feedback. Together with the spatial and cell-type-specific enrichment of MEIS3 observed in **Figure 6**, these results support the hypothesis that MEIS3 may orchestrate a stromal cytokine regulatory network within MSCs. However, given the very modest correlation coefficients and limited sample size, these findings should be interpreted as preliminary, reflecting subtle associations rather than strong linear relationships, and warrant validation in larger cohorts.

### 3.9 MEIS3-related ceRNA regulatory network in HCM

**Figure 8:**
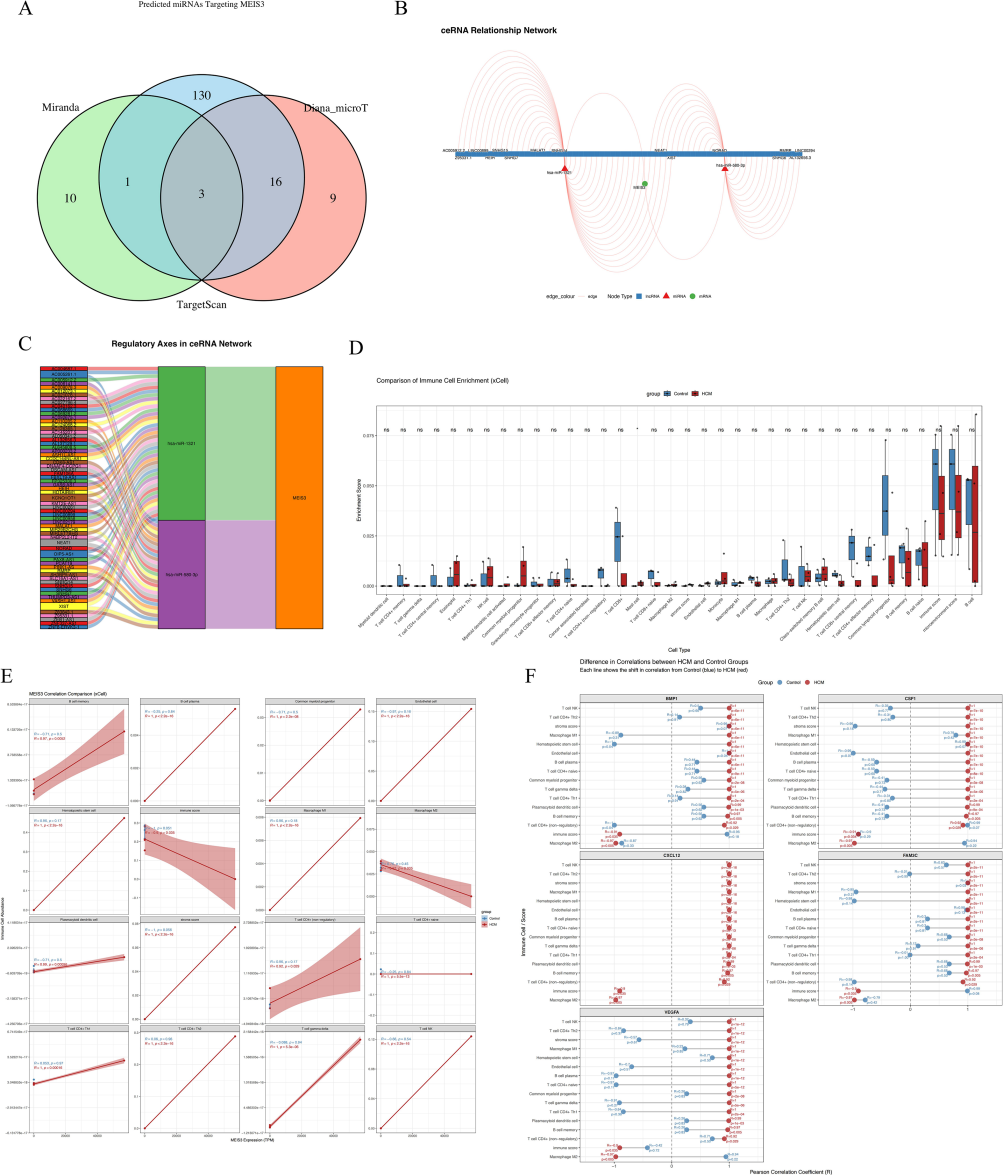
Construction of MEIS3-related ceRNA regulatory network and immune cell infiltration landscape in HCM. **(A)** Venn diagram displaying the intersection of predicted miRNAs targeting MEIS3 from three databases (miRanda, TargetScan, and Diana_microT), identifying 3 common miRNAs. **(B)** ceRNA network visualization including lncRNAs, miRNAs, and MEIS3, with red triangles indicating lncRNAs, green circles indicating miRNAs, and blue squares representing mRNAs. Regulatory edges highlight potential competing interactions. **(C)** Regulatory axis diagram illustrating the ceRNA pathway of MEIS3, showing multi-layered lncRNA–miRNA–mRNA relationships. Notably, MEIS3 is positioned as a central node downstream of multiple axes. **(D)** Immune cell enrichment analysis using xCell revealed significantly altered immune/stromal cell populations in HCM compared to controls. Notably, fibroblasts, endothelial cells, macrophages M1/M2, and dendritic cells (aDCs, pDCs) displayed robust enrichment differences (Wilcoxon test, p < 0.05). **(E)** MEIS3 expression exhibited statistically significant correlations with selected immune cell types in the HCM group, especially macrophages, dendritic cells, and endothelial cells. Shaded bands represent 95% confidence intervals (Spearman correlation, p < 0.05). **(F)** Correlation heatmap illustrating disease-specific associations between five representative cytokines (BMP1, CSF1, CXCL12, FAM3C, VEGFA) and differentially enriched xCell-derived immune cell types in the HCM group. Distinct patterns were observed for cytokine–cell type relationships, with stronger or inverse correlations in HCM, suggesting disease-specific immune remodeling.

To investigate upstream mechanisms underlying MEIS3 dysregulation in HCM, we constructed a competing endogenous RNA (ceRNA) network by integrating predictions from miRanda, TargetScan, and Diana_microT. Cross-database comparison identified three shared miRNAs predicted to target MEIS3 (**Figure 8A**). These served as the basis for building a MEIS3-centered ceRNA network incorporating lncRNAs and miRNAs (**Figure 8B**). The resulting network displayed a dense regulatory architecture, suggesting that multiple lncRNAs may act as molecular sponges to modulate MEIS3 levels by sequestering its targeting miRNAs. A Sankey diagram (**Figure 8C**) highlights several candidate regulatory axes, including AC009403.1–miR-129-5p–MEIS3 and SNHG16–miR-335-5p–MEIS3. These findings support a model in which ceRNA-mediated post-transcriptional regulation contributes to MEIS3 overexpression in HCM cardiac tissue, providing potential new nodes for mechanistic exploration and therapeutic targeting.

### 3.10 MEIS3-associated immune remodeling via stromal–cytokine interactions

We evaluated the immune–stromal milieu using xCell deconvolution of bulk RNA-seq. Relative to controls, HCM samples showed higher enrichment scores for fibroblasts, endothelial cells, macrophages (M1/M2), and dendritic cells, with lower CD8^+^ T cell and NK cell signals, consistent with stromal activation and an altered immune landscape (**Figure 8D**). Associations between MEIS3 expression and cell-type scores indicated positive correlations with fibroblasts, endothelial cells, macrophages, and aDCs in HCM (but not in controls), suggesting disease-specific immune–stromal coupling (**Figure 8E**). To examine potential cytokine mediators, we correlated MEIS3-linked cytokines with immune/stromal scores. CXCL12, VEGFA, BMP1, and CSF1 showed cell-type–specific correlation patterns with macrophages, T cells, and endothelial subsets (**Figure 8F**, **Supplementary Figure S2**). Taken together, these observations are consistent with a cytokine-mediated stromal–immune axis associated with MEIS3 in HCM. Given that several effect sizes are modest, we interpret these findings as hypothesis-generating; validation in larger cohorts and functional assays will be required to determine whether MEIS3 causally modulates the cardiac immune microenvironment.

## 4 Discussion

In this study, we integrated bulk and single-cell transcriptomics with computational modeling to explore a potential link between the developmental transcription factor MEIS3 and the immunopathology of hypertrophic cardiomyopathy. Our key finding is that MEIS3 is significantly upregulated in HCM hearts and that its elevated expression is associated with distinct immunological and clinical features of the disease. MEIS3 stood out in our unbiased multi-omics analyses as a candidate diagnostic biomarker (in our dataset) and as a putative immunoregulatory switch in the HCM myocardium. To our knowledge, this is among the first report implicating MEIS3 in HCM. Given the modest sample size and cross-dataset integration, these observations should be regarded as hypothesis-generating. More broadly, they add to evidence that HCM involves mechanisms beyond sarcomeric gene mutations (20, 21), suggesting how transcriptional regulators and immune mechanisms may converge to drive disease progression.

Several lines of evidence from our results underscore the clinical relevance of MEIS3 in HCM. First, MEIS3 was one of the top differentially expressed genes in HCM, with a fold-change and significance that suggest a robust disease association in this cohort. This alone is notable given that prior transcriptomic studies of HCM have not reported MEIS3, possibly because it may not reach significance in larger heterogeneous cohorts or was simply overlooked. The pronounced upregulation in our well-phenotyped cohort may reflect a specific subset of HCM where MEIS3 is important, or fundamental involvement in HCM that warrants confirmation in larger samples. Second, our machine learning analyses identified MEIS3 as a powerful single-gene classifier for HCM. In our discovery dataset, the AUROC exceeded 0.90, and in an independent myocardial cohort the discriminatory performance remained high (AUC ~0.85), on par with or exceeding several multi-gene signatures proposed previously (22, 23). This suggests that measuring MEIS3 (for instance, via PCR on endomyocardial biopsy or circulating blood cells) could aid in HCM diagnosis or in distinguishing HCM from other causes of hypertrophy. It is particularly intriguing to consider MEIS3 as a biomarker in genotype-negative HCM patients, where traditional genetic testing fails to provide a diagnosis. Our work lays the groundwork for future studies to validate MEIS3 in a larger patient population and assess its additive value to current diagnostic algorithms.

Consistent with prior evidence, peripheral blood transcriptomes can partially mirror myocardial remodeling and clinical outcomes, supporting the concept that circulating immune cells provide an accessible—though indirect—window into cardiac disease activity (24, 25). In the context of MEIS3, two non-exclusive models may explain the concordance of blood- and tissue-derived signals. One is a cardiac-intrinsic model, in which MEIS3 acts within resident stromal cells to regulate fibrotic and immunomodulatory pathways (26). The other is a systemic/indirect model, whereby circulating immune cells—such as CCR2^+^ monocytes/macrophages—are recruited from blood to injured myocardium and influence local remodeling (27). As our study lacks paired blood–tissue samples, we frame such cross-compartment inferences as hypothesis-generating rather than confirmatory. We explicitly acknowledge this limitation and outline next steps, including paired sampling, single-cell/spatial localization of MEIS3 in myocardium, and prospective validation in independent HCM cohorts. Finally, given that observed correlations (e.g., with cytokines) are statistically significant but very weak in magnitude, they should be regarded as biologically tentative until replicated in larger datasets.

Beyond diagnosis, the immunological role of MEIS3 in HCM is a working hypothesis with mechanistic implications that requires further testing. High MEIS3 expression was linked to fibroblast activation, altered cytokine profiles (e.g., CXCL12, VEGFA, BMP1), and reduced immune infiltration, but the correlations were very weak (e.g., MEIS3–CXCL12 r ≈ 0.06), indicating subtle rather than robust effects. Similar to observations in oncology where MEIS family members mark immune exclusion and “cold” tumors (19, 28), these findings suggest that MEIS3 may contribute to feedback circuits that dampen inflammation while promoting fibrosis. Such statistically significant but weak correlations are common in single-cell data, where large cell numbers inflate significance and heterogeneity, paracrine gradients, or non-linear dynamics can mask stronger local interactions. Thus, the observed associations should be regarded as preliminary indicators of complex immune–stromal regulation rather than direct linear relationships. By analogy—and explicitly as a hypothesis—we speculate that in HCM, MEIS3 might contribute to an immune-modulatory feedback loop that restrains excessive inflammation but perhaps at the cost of promoting fibrotic remodeling. Potential mechanisms include regulation of PDK1/Akt signaling, which influences macrophage polarization and fibroblast survival (29), and interactions with suppressive cytokines such as TGF-β or IL-10. Indeed, our data showed that samples with high MEIS3 and stromal growth factors had lower T cell and macrophage infiltration. It is tempting to consider that MEIS3 could be driving expression of factors that dampen immune cell recruitment or activation in the myocardium. Although direct targets of MEIS3 in the heart are unknown, candidates might include genes involved in chemokine signaling or antigen presentation. Future chromatin immunoprecipitation sequencing (ChIP-seq) for MEIS3 in cardiac cells could illuminate its gene regulatory network. Future work using perturbation assays and ChIP-seq in cardiac stromal cells is needed to test these hypotheses.

Our single-cell analysis further identified fibroblasts and vascular cells as the principal sources of MEIS3 in HCM. Cardiac fibroblasts are central players in myocardial remodeling that not only produce but also respond to cytokines, and elevated MEIS3 may enhance their proliferative and matrix-secreting capacity while modulating crosstalk with immune cells. For instance, CXCL12, which we found enriched in stromal compartments, is typically secreted by fibroblast-like cells and can recruit progenitors or regulate T cell responses. Increased MEIS3 may therefore promote CXCL12-dependent retention of CXCR4^+^ progenitors or regulatory T cells, conferring fibroblasts with an immunoregulatory phenotype that supports chronic low-grade inflammation in HCM. In contrast, cardiomyocytes displayed minimal MEIS3 expression, suggesting that any hypertrophic effects are likely mediated indirectly through stromal intermediaries. This highlights the importance of analyzing cell–cell interactions in HCM; what has traditionally been viewed as a disease of cardiomyocytes may significantly involve fibroblast-immune cell networks modulated by factors like MEIS3.

Our ceRNA network analysis provides a preliminary hypothesis for upstream regulation of MEIS3 in HCM. We identified specific lncRNAs that could act as sponges for MEIS3-targeting miRNAs, potentially explaining why MEIS3 is overexpressed. Notably, these lncRNAs are largely uncharacterized in cardiac contexts. If validated, they might represent novel regulatory nodes that coordinate with MEIS3 in driving disease. It is intriguing to consider that genetic or epigenetic changes in non-coding regions (rather than coding mutations) might contribute to HCM in some patients by dysregulating gene networks like the MEIS3 hub. This aligns with a growing recognition that non-sarcomeric and regulatory elements can influence HCM severity (30, 31). Clinically, such lncRNAs could become therapeutic targets: silencing a disease-promoting lncRNA might restore microRNA activity to normal and thereby reduce MEIS3 levels and its downstream effects.

Our findings suggest that MEIS3 may contribute to HCM pathogenesis and hold potential clinical relevance. As a biomarker, MEIS3 expression could assist in early detection or risk stratification, particularly in patients with borderline hypertrophy or a family history without known mutations. Beyond diagnosis, MEIS3 and its regulatory network may represent candidate therapeutic targets, an area of unmet need given the absence of disease-modifying therapies for HCM. Although direct inhibition of transcription factors is challenging, indirect strategies—such as disrupting MEIS3–DNA binding or targeting upstream regulators like lncRNAs and signaling pathways—merit exploration. The link between MEIS3 and PI3K–Akt signaling is especially noteworthy, as pharmacological modulators of this pathway already exist. Nonetheless, these therapeutic implications remain hypothesis-generating; proposals to repurpose anti-fibrotic or immunomodulatory agents (e.g., TGF-β modulators, IL-1β antagonists) should be viewed as speculative until supported by rigorous mechanistic validation. Importantly, while high MEIS3 expression was associated with a distinct cytokine milieu, correlation does not establish causality, underscoring the need for functional and prospective studies to test whether MEIS3-related pathways can be therapeutically modulated to improve outcomes.

More broadly, this work supports the concept that HCM, traditionally regarded as a purely genetic cardiomyopathy, also encompasses an immunological dimension. This aligns with recent reviews highlighting inflammation as a modifier of HCM phenotypes (32, 33). By integrating immunology and cardiology through the lens of MEIS3, we provide a framework for future investigations to explore how immune cells and heart cells co-act in HCM. As techniques like single-cell sequencing and spatial transcriptomics become more prevalent, we anticipate that additional factors like MEIS3 will be uncovered, further blurring the line between classic genetic paradigms and immune-mediated processes in cardiomyopathy. In conclusion, our work identifies MEIS3 as a candidate player in HCM and encourages a re-examination of HCM therapeutic strategies to include modulation of immune and gene regulatory networks. Future priorities include replication in external cohorts, paired blood–tissue profiling, spatial localization, and perturbation studies to test causality. Targeting the MEIS3-centered network might offer a two-pronged benefit: attenuating pathological cardiac remodeling and recalibrating the cardiac immune response, ultimately improving outcomes for patients with this complex disease.

## 5 Limitation

Our findings should be interpreted in light of certain limitations. The sample size of our study was modest (n=7), reflecting the difficulty in obtaining myocardial tissue from HCM patients and matched healthy controls. While this was sufficient for our multi-omics pilot analysis, larger cohorts are needed to verify the consistency of MEIS3 upregulation and to refine its correlation with immune features. The extreme correlations observed (e.g., CXCL12 with stromal score) likely stem from low sample numbers and should be validated with caution. Another limitation is that our study is primarily associative. We demonstrate correlations between MEIS3 and immune parameters, but this does not prove causation. It remains to be experimentally shown whether MEIS3 actively drives immune modulation in HCM or is simply a bystander. *In vitro* and *in vivo* studies will be crucial next steps – for example, overexpressing or knocking down MEIS3 in cardiac fibroblasts to observe effects on cytokine production, or using a mouse model of cardiac hypertrophy to test if MEIS3 loss-of-function alters inflammatory cell infiltration and fibrosis. Additionally, while we did leverage an external dataset for diagnostic validation, a comprehensive external validation (including protein-level confirmation such as immunohistochemistry for MEIS3 in HCM myocardium) is warranted. Despite these limitations, our integrative approach provides a coherent narrative that links MEIS3 to known HCM pathways (fibrosis, immune response) and generates novel hypotheses.

## Data Availability

The original contributions presented in the study are publicly available. This data can be found here: ArrayExpress, E-MTAB-15843.
