# Supplementary material for: Integrative multi-omics identifies MEIS3 as a diagnostic biomarker and immune modulator in hypertrophic cardiomyopathy
Source: Front Immunol. 2025 Oct 22;16:1675467. doi: 10.3389/fimmu.2025.1675467 (PMC12586186; doi:10.3389/fimmu.2025.1675467)
Supplement: Supplementary file 1 [file DataSheet1.docx]

**Supplementary Figure**


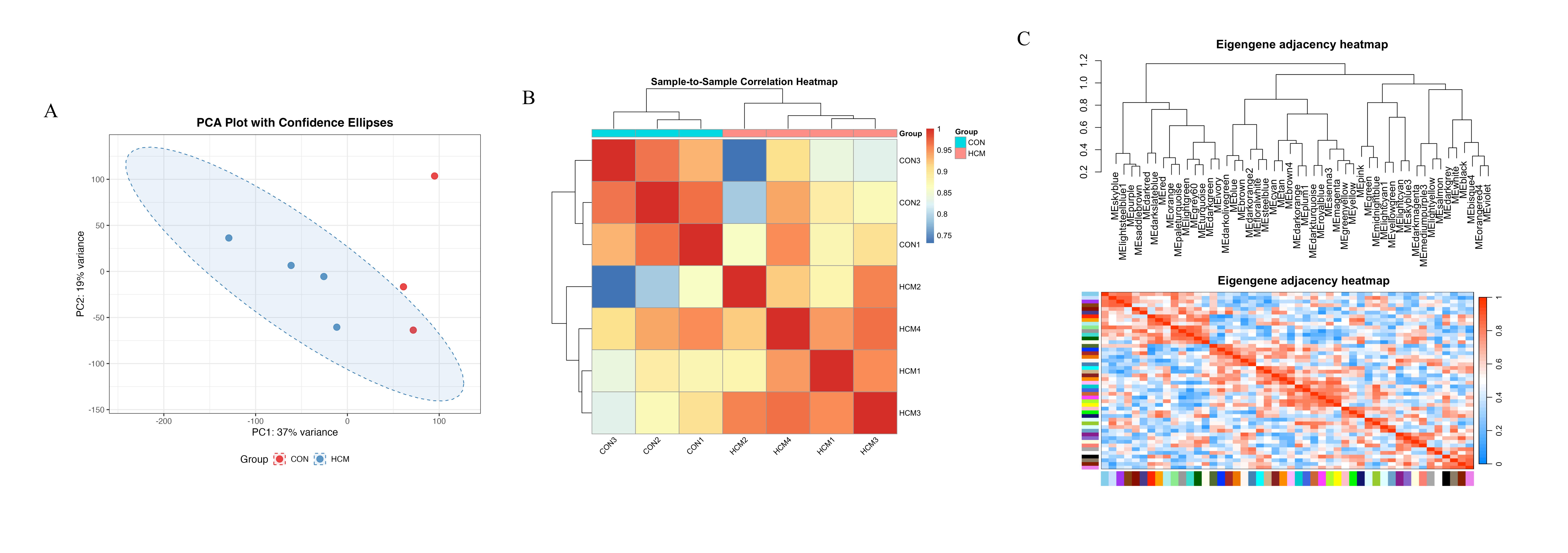


**Supplementary Figure S1. Sample-level quality control and WGCNA module topology.**

**(A)** Principal component analysis (PCA) plot showing distinct separation between HCM (red) and control (blue) samples. PC1 explains 37% and PC2 explains 19% of total variance. Confidence ellipse highlights inter-group separation.

**(B)** Sample-to-sample correlation heatmap. Warmer colors (red) represent stronger pairwise Pearson correlations. Dendrogram shows unsupervised clustering; control samples (CON1–3) and HCM samples (HCM1–4) form distinct branches.

**(C)** Top: Dendrogram of module eigengenes showing hierarchical clustering of co-expression patterns among modules. Bottom: Heatmap of eigengene adjacencies, where red indicates strong correlation between modules and blue indicates weak similarity. Color bars indicate WGCNA module identity.


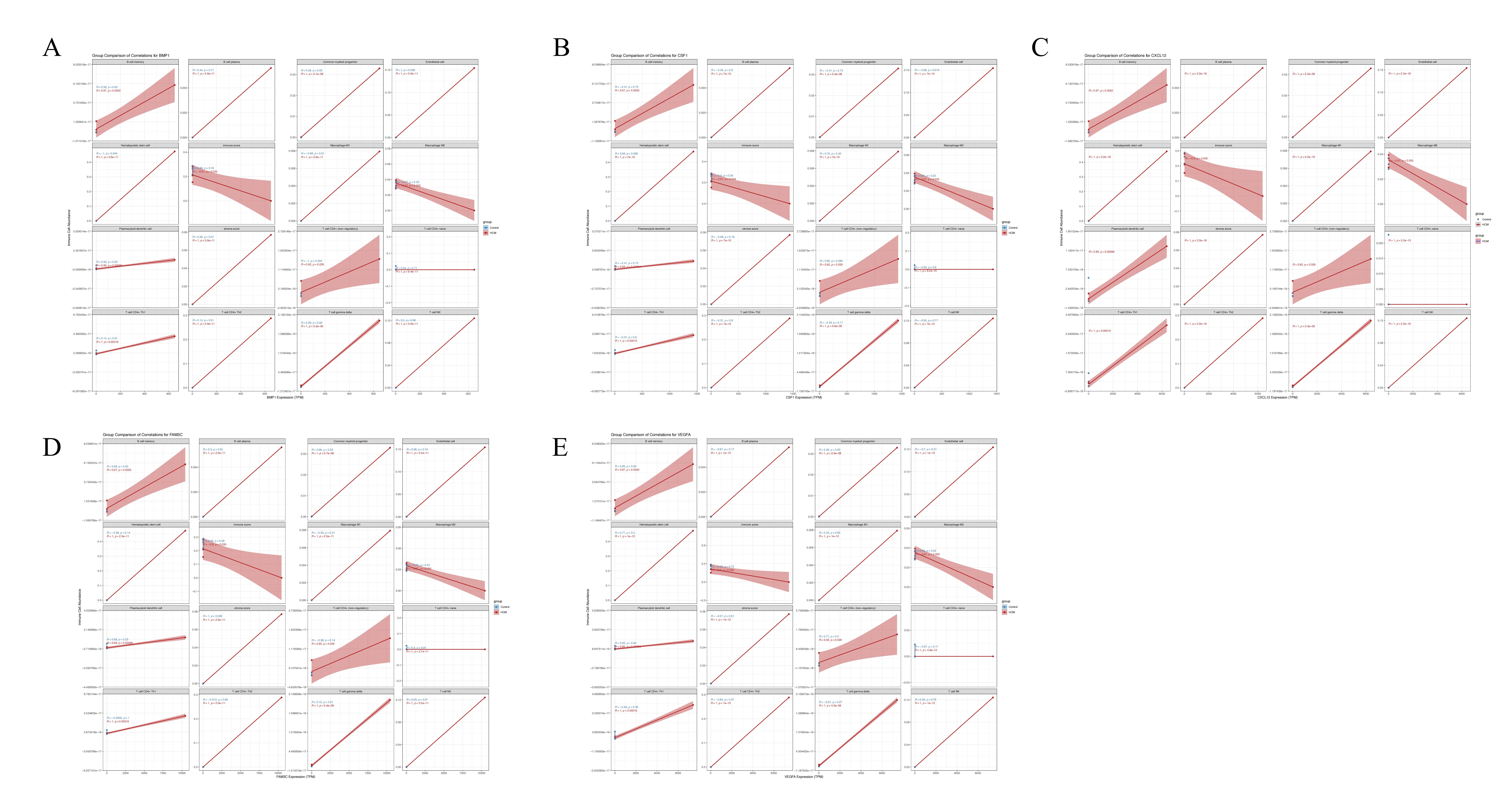


Supplementary Figure S2. Expanded cytokine–immune correlation analysis across HCM-enriched cell types.

(A–E) Grouped correlation scatter plots depicting expression-based associations between BMP1 (A), CSF1 (B), CXCL12 (C), FAM3C (D), and VEGFA (E) and cell type-specific enrichment scores across five immune/stromal populations: endothelial cells, fibroblasts, macrophages, T helper 1 cells, and Tregs. Regression slopes and significance values (p) are indicated per group (HCM in red, control in blue). These cytokines displayed cell-type dependent differential associations under HCM conditions, further supporting cytokine-mediated immune microenvironment remodeling.
